# Incremental Improvements Can Reduce Alarm Fatigue in the Neonatal Intensive Care Unit

**DOI:** 10.1097/pq9.0000000000000399

**Published:** 2021-02-12

**Authors:** Amy B. Schlegel, Edward G. Shepherd

**Affiliations:** From the * The Ohio State University College of Medicine; † Division of Neonatology, Department of Pediatrics, Nationwide Children’s Hospital, Columbus, Ohio

High-level, high-acuity neonatal intensive care for extremely premature infants and critically ill newborns requires complex, technologically advanced devices including incubators, mechanical ventilators, extracorporeal membrane oxygenation circuits, physiologic monitors, and life-sustaining medication infusions. In turn, each of these devices has multiple failure modes that must be carefully monitored, tracked, and attenuated, usually via alerts and alarms. The overall result of all these technological and scientific advances is today’s modern neonatal intensive care unit (NICU). This miraculous place can sustain the life of a 400-g, 22-week infant for months on end while they complete what should have been a 40-week gestation. However, one of the unintended consequences of this miracle is an ever-growing cacophony of disruptive alarms and notifications. We rely on these alerts and notifications to ensure that temperatures, oxygen saturations, blood pressures, respiratory patterns, cerebral and organ perfusion, and heart rates remain within normal parameters. This technology and monitoring gives providers real-time awareness of acute changes in patient stability or device function, thereby allowing timely intervention to avoid life-threatening injury.

Only recently have we become aware that the sum of all of these alarms and alerts is so overwhelming that caretakers routinely suffer “alarm fatigue.” The most insidious result of this fatigue is that caretakers ignore all alerts, leading to potential patient harm.^1,2^ Furthermore, high alarm burden and alarm-related noise in the NICU contribute to nursing burnout, parental distress, and an adverse neurodevelopmental environment for neonates.^3–5^ But is alarm fatigue merely the price of doing business in a modern NICU?

Drs. Fang, McCauley, and colleagues take on this critical question in their important QI report “Reducing Alarm Burden in a Level IV Neonatal Intensive Care Unit,” which cleverly applies the thoughtful use of technology itself to tackle the challenge of reducing alarms and alerts in a high-acuity NICU. Using data-driven QI methodology, the authors capitalized upon the archiving capabilities of vital sign monitors by efficiently using objective, accurate, detailed data to identify self-resolving alerts of little clinical significance while noting the normal fluctuations of oxygen saturation as high-yield opportunities for improvement. Surprisingly, the team observed that such alarms occurred an average of 14 times per hour within this patient population, or once every 4 minutes, 24 hours/d. Consequently, it is not unexpected that bedside staff often tune these alarms out or turn them off entirely.

The team created a series of carefully constructed, iterative Plan-Do-Study-Act cycles utilizing stepwise changes in vital sign monitor parameters to test potential improvements. Each of these changes was implemented and evaluated via both direct observation and archived data. Then, each evaluation was examined to inform subsequent cycles and to fine-tune the interventions further. Importantly, staff were surveyed before and after the project to determine their subjective perception of alarm fatigue, and the investigators carefully monitored appropriate oxygen saturations as a balancing measure.

The team’s QI efforts led to an impressive 64% reduction in alarms per hour, far surpassing their 50% decrease goal. This result demonstrates that the device’s intrinsic capabilities in a standardized fashion effected change in their NICU. This improvement has been sustained mainly due to hard-wired changes that automatically minimize the burden of alerts, not requiring intervention to improve patient well-being.

Notably, the authors ensured ongoing patient safety by demonstrating that, despite reducing self-resolving alarms, the studied patients did not experience a change in average time spent within their targeted oxygen saturation range. Oxygen saturation targeting is critically important in the NICU to avoid various negative outcomes associated with hyperoxia and hypoxia in extremely preterm infants.^6^

The sound methodology, objective technology-based data collection, and hard-wired interventions leading to compliance with practice change make this report a vital contribution to the literature. Similarly, these strengths make this team’s multidisciplinary effort potentially applicable to level IV NICUs elsewhere. This QI report focuses its initiative on a homogenous population of neonates requiring supplemental oxygen. It excludes patients with hypoxic respiratory failure complicated by congenital heart disease or pulmonary hypertension as target SpO2 parameters in this more heterogeneous population have greater interpatient variability. Nonetheless, the applicability of this report should be viewed as broad enough to apply to the safety and standardization of care efforts in any level IV unit. With thoughtful, data- and evidence-driven initiatives, these stepwise efforts aimed at reducing self-resolving alarms can be targeted to specific patient needs and parameters.

The authors should be congratulated on successfully undertaking an initiative that has led to impactful, sustainable change in their level IV NICU. Their work has contributed to creating a safer NICU patient-care environment that fosters positive neurodevelopment, facilitates parental mental-health, supports staff well-being, and should be considered by other multidisciplinary NICU teams as a model for reduction of alarm burden. The biggest challenge that remains is the fact that even after a 64% reduction in self-resolving alarms, they still occur every 10 minutes. Clearly, we still have work to do.

## DISCLOSURE

The authors have no financial interest to declare in relation to the content of this article.

## References

[1] Johnson KR Hagadorn JI Sink DW. Alarm safety and alarm fatigue. Clin Perinatol. 2017; 44:713–728 28802348 10.1016/j.clp.2017.05.005

[2] Joint Commission. Medical device alarm safety in hospitals Sentinel Event Alert. 2013; 50:1–3 23767076

[3] Graham KC Cvach M. Monitor alarm fatigue: standardizing use of physiological monitors and decreasing nuisance alarms. Am J Crit Care. 2010; 19:28–34; quiz 35 20045845 10.4037/ajcc2010651

[4] Topf M Dillon E. Noise-induced stress as a predictor of burnout in critical care nurses. Heart Lung. 1988; 17:567–574 3417467

[5] Almadhoob A Ohlsson A. Sound reduction management in the neonatal intensive care unit for preterm or very low birth weight infants. Cochrane Database Syst Rev. 2020; 1:CD010333 31986231 10.1002/14651858.CD010333.pub3 PMC6989790

[6] Askie LM Darlow BA Finer N; Neonatal Oxygenation Prospective Meta-analysis (NeOProM) Collaboration. Association between oxygen saturation targeting and death or disability in extremely preterm infants in the Neonatal Oxygenation Prospective Meta-analysis Collaboration. JAMA. 2018; 319:2190–2201 29872859 10.1001/jama.2018.5725 PMC6583054

